# Climate change increases the number of landslides at the juncture of the Alpine, Pannonian and Mediterranean regions

**DOI:** 10.1038/s41598-023-50314-x

**Published:** 2023-12-27

**Authors:** Mateja Jemec Auflič, Nejc Bezak, Ela Šegina, Peter Frantar, Stefano Luigi Gariano, Anže Medved, Tina Peternel

**Affiliations:** 1 Geological Survey of Slovenia, Dimičeva ulica 14, 1000 Ljubljana, Slovenia; 2 Faculty of Civil and Geodetic Engineering, University of Ljubljana, Jamova cesta 2, 1000 Ljubljana, Slovenia; 3 Slovenian Environmental Agency, Vojkova 1b, 1000 Ljubljana, Slovenia; 4 CNR-IRPI (Italian National Research Council), Via Madonna Alta, 126, 06128 Perugia, Italy

**Keywords:** Natural hazards, Climate-change impacts

## Abstract

During the next few decades, changes in rainfall frequency and magnitude are expected to have major impacts on landscape evolution, social, and economic aspects of human society. We focus on seasonal rainfall variations by the end of the twenty-first century to define affected landslide-prone areas, future landslide alerts and the impact of landslides on landscape development in the juncture of the Alpine, Pannonian, and Mediterranean region. A moderate and a worst-case climate scenario from CMIP5 global climate simulations were considered to determine the impact of rainfall on the two most common types of landslides in region, shallow and deep-seated landslides. The observed changes in the occurrence of shallow landslides are significant, especially in the winter months, where we can expect more landslide-prone areas compared to the baseline period. Shallow landslides will have a greater impact on the landscape in spring and summer than deep-seated landslides, especially in vineyards.

## Introduction

Landslides are considered one of the factors that shape the Earth’s surface, especially over long time scales, by transfer of mass by denudation of upland areas and sedimentation in valley floors and sedimentation basins^1,2^. Slope stability conditions are influenced by several factors, among which rainfall—mostly rainfall—is by far the most relevant. The ongoing and projected changes in the frequency and intensity of rainfall events due to global warming are expected to modify the frequency, abundance, and distribution of landslides of different types^3^. Moreover, land cover/use changes may affect landslide occurrence or activity either directly or indirectly^4^. However, quantifying the extent and the magnitude of all these changes is a tricky task. Simple inferences (i.e., higher rainfall intensity—higher landslide risk) can be erroneous since hydro-geo-morphological nuances determine the trajectory of changes in landslide behavior^5^. Quantitative, regional-scale analyses of the impact of projected rainfall variations on landslides of various types and in different physiographical settings are necessary to obtain reliable results and reduce all the inherent uncertainties.

Several authors have investigated the possible link between climate change and landslides at different temporal and spatial scales^3,5^. Overall, around 150 peer-reviewed articles are currently included in the scientific literature, mostly concentrated in Europe. Recently, regional-scale analyses of the impact of projected changes in rainfall features on landslide occurrence were carried out in Austria^6,7^, Italy^8–10^, Taiwan^11^, Canada^12,13^, China^14,15^, France^16^, Portugal^17^ and for European Alps^18^ and European land transport infrastructure^19^. Studies on future variations of land cover/use were also conducted, mostly aiming at evaluating the impact of such changes on landslide susceptibility^6,20–22^.

**Figure1:**
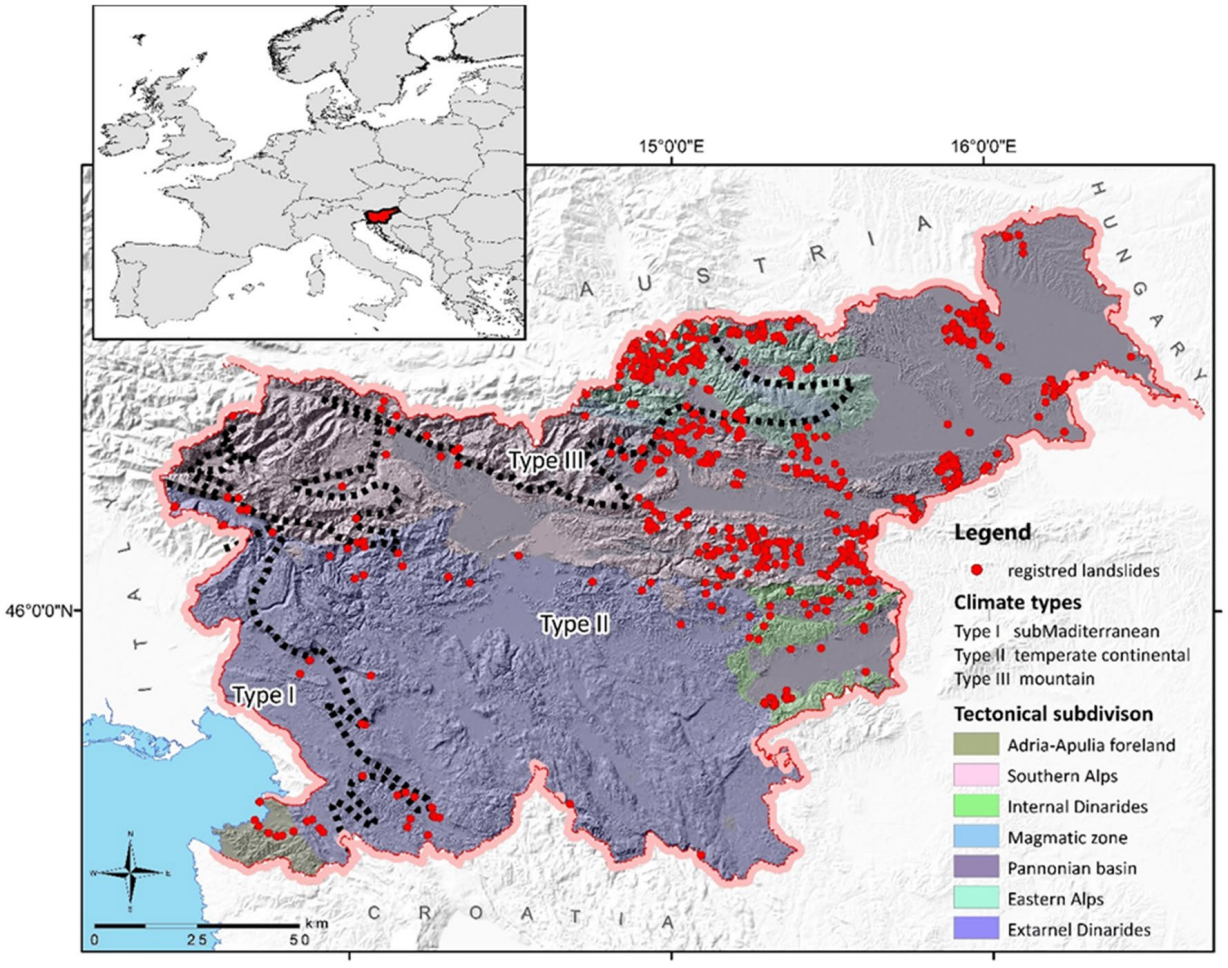
Map of Slovenia with climate types, tectonic subdivisions, and location of registered landslides in Slovenia.

Slovenia as one of the countries with a high susceptibility to landslide occurrence^23^ is also characterized by three different climate zones^24^ at the junction of the Alps, the Pannonian Plain, the Dinaric Mountains, and the Mediterranean region (Fig. 1). Despite its relatively small size (i.e., around 20,000 km^2^) and interesting case study that can generate findings useful also for neighbouring countries or the similar environment around the globe. The effects of climate change on the landscape are evident in the increasing landslides triggered by severe summer storms, while the prolonged precipitation typical for the nineteenth and twentieth centuries has become less frequent in the twenty-first century. Komac^25^ was one of the first in Slovenia to analyse the importance of land cover for slope instability. He considered land cover as one of the input data for susceptibility map^26^. Since the Slovenian national landslide forecasting system (MASPREM) has been in operation since 2013, we also systematically record the location of the landslide, the date of the trigger, and the type of land cover in the landslide source. Based on this information, Jemec Auflič et al.^27^ showed a strong correlation between shallow landslides and land cover which were also highlighted by numerous authors^4,28,29^. Despite the relatively small soil volume involved, the shallow landslides can be densely distributed over the areas and shape the landscape over time through superficial processes^30^. In contrast, deep-seated landslides are a combination of two or more landslide types^31^ with a relatively deeper sliding surface, a lower velocity, and a slide volume that often does not slide completely down the slope at once; rather, some of the slide volume remains on the slope^32,33^. The response of these two types of landslides to climate change is expected to be different, given that shallow, rapid landslides are controlled by rainfall peaks and by rainfall intensity at short durations, whereas deep-seated, slow-moving landslides are conditioned chiefly by long-term (e.g. monthly or seasonal) cumulated rainfall, and the related groundwater variations^34,35^.

**Figure 2:**
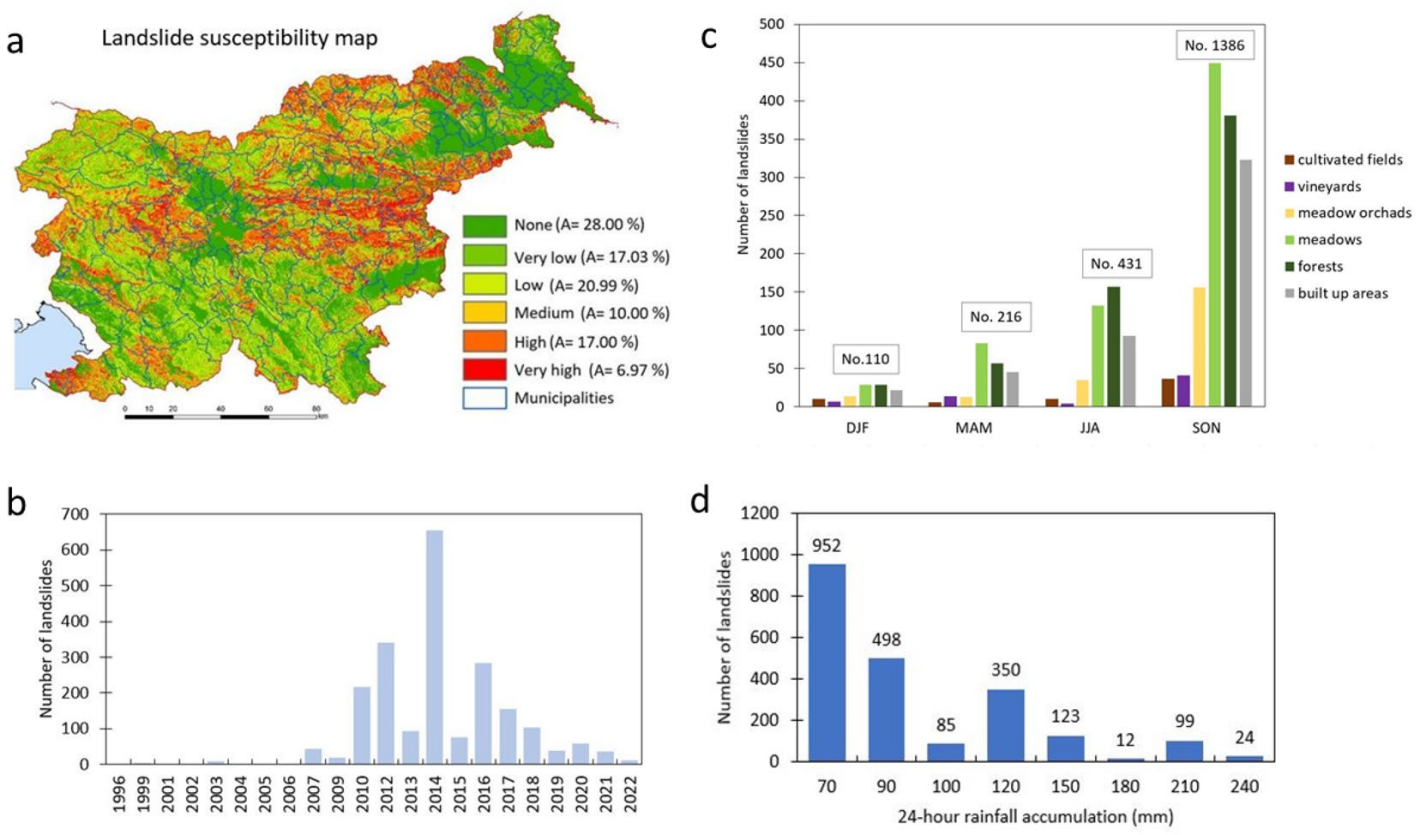
(**a**) Landslide susceptibility map^26^ with proportion of the area, covered by given class (A). (**b**) Number of landslides per year. (**c**) number of landslides per season per land cover classes for the period between 1996 and 2022. Seasons are labelled DJF (December, January, February), MAM (March, April, May), JJA (June, July, August), and SON (September, October, November). (**d**) 24-h rainfall accumulation responsible for landslides triggering.

Studies so far showed that one-third of Slovenia is highly susceptible to landslides and almost one-fifth of the Slovenian population lives in areas that are highly prone to landslides^26,36^. The most common phenomena in Slovenia are shallow landslides, which are mainly caused by intense short- or long-term rainfall events^36–40^. In the past 25 years, more than 10,000 landslides have been recorded by the Administration for Civil Protection and Disaster Relief (ACPDR) and the Geological Survey of Slovenia (GeoZS). Among the many slides that have been recorded are those landslides whose volumes exceeded one million cubic meter and which, in addition to the damage done to buildings, also endangered the lives of hundreds of people and even resulted in human casualties^41^. These failures correspond to deep-seated landslides^32^, in which unstable masses develop into debris flow. In all cases, the sliding occurred in clastic rocks (mostly flysch) and located below steep slopes composed of highly permeable carbonate rocks. Due to faults and cracks in the surface rocks, a large part of rainfall is able to penetrate and supply the groundwater recharge in the weathered clastic zones. Figure 2a shows the number of landslides per year in 1996 and 2022 with known locations, while Fig. 2b shows the distribution of landslides by season and land cover (Corine land cover data from 2018). However, due to anticipated climate change, the frequency and intensity of rainfall events are expected to increase, thus impacting the occurrence of landslides in Slovenia^36^.

In this paper, we focus on the study of the impact of future seasonal rainfall variations on landslides occurrences by the end of the twenty-first century. We employ a moderate and a worst-case climate scenario (i.e., RCP4.5 and RCP8.5), using the rainfall variable as one of the most common landslides triggering factors, to answer the following questions: (1) How does future seasonal variability in rainfall affect landslide-prone areas (i.e. shallow landslides and groundwater recharge, as a proxy for triggering deep-seated landslides)? (2) How many landslide alerts might we expect in the future? (3) How anticipated climate change impact on land cover change?

## Results

### Seasonal variability of rainfall on landslide-prone areas

**Figure 3:**
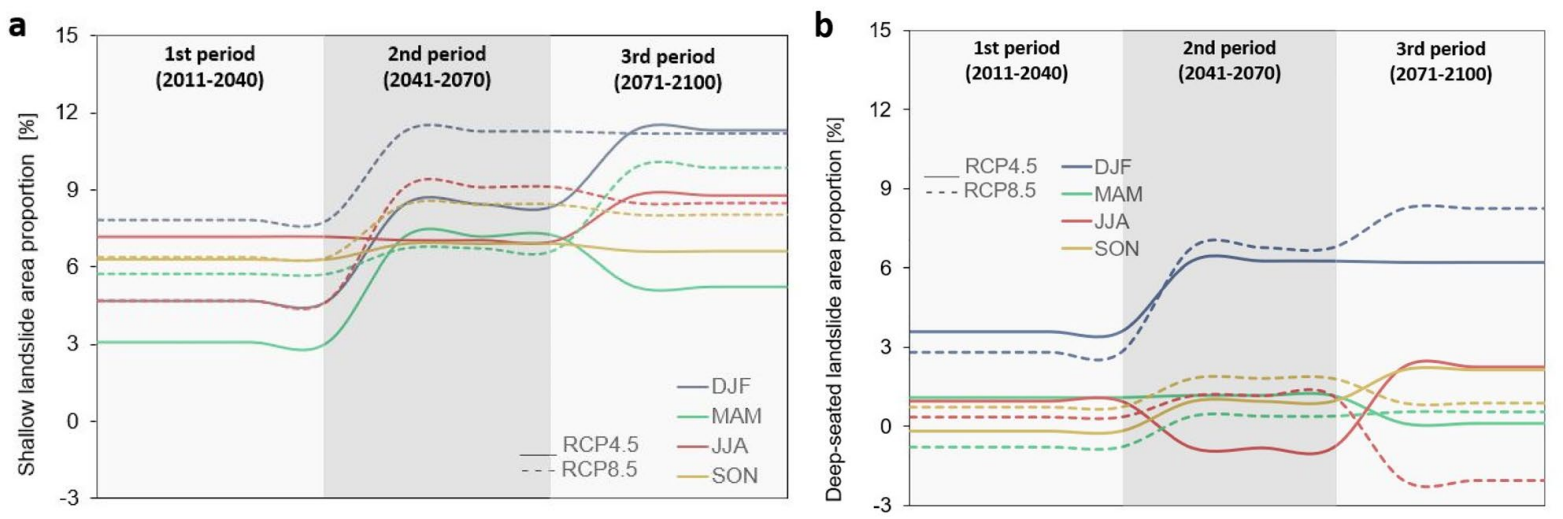
Variability of shallow landslide proportion area and deep-seated landslide in percent (%) between seasonal median values of baseline (1981–2010) and projection periods (1st period-near term, 2nd period-mid-century, 3rd period-end of the twenty-first century) for both RCP 4.5 and RCP 8.5 scenarios. Values indicate the 50th percentile of all six climate models. (**a**) Comparison of seasons refers to landslide-prone areas derived from the MASPREM algorithm. (**b**) Comparison of seasons refers to groundwater recharge which could indicate deep-seated landslides derived from the mGrova algorithm. Seasons are labelled DJF (December, January, February), MAM (March, April, May), JJA (June, July, August), and SON (September, October, November).

A first analysis compares seasonal variability of rainfall between the baseline period and three projection periods (near-term, mid-century and end of the century) on landslide-prone areas calculated based on the MASPREM algorithm (Fig. 3a) and water recharge derived from the mGrova model (Fig. 3b) both for RCP 4.5 and RCP 8.5 scenarios. In both Fig. 3a and 3b we considered all three landslide susceptibility classes (LSC; i.e. low–medium–high) as a significant value for landslide-prone areas. Specifically, we proceeded from the assumption that landslides can occur in all classes, with the difference that the class determines where the probability of landslides occurrence is higher. We plotted the 50th percentile values because there are minor differences between the 25th and 75th percentile values. Positive values in the graphs of Fig. 3a indicate that shallow landslide-prone areas will increase over the future projection periods, while negative values indicate that the percentage of shallow landslide-prone areas will decrease. Similarly, positive values in Fig. 3b indicate that groundwater recharge will increase, which could indicate an increase in the probability of the deep-seated landslide formation.

The trend in seasonal variability of landslide-prone areas (Fig. 3a) increases from the first to the third period for RCP4.5, except for the spring months, for which the models predict a lower percentage of landslide-prone areas than at mid-century. For instance, the trend in winter, summer, and autumn months shows a gradual increase in landslide-prone areas from 5 to 12% from the first to the third period. In the case of climate scenario RCP8.5, the largest changes are expected for winter, when up to 12% more landslide-prone areas are expected in the second and third periods compared to the baseline period. In spring, the landslide-prone area increases from the first to the third period, and it is projected that about 10% of the country could be landslide-prone by the end of the century. Seasonal variations in landslide-prone areas are also significant for the summer and autumn months, especially at mid-century, while projections for the end of the century do not show a higher percentage of landslide-prone areas than at the mid-century.

Changes in the groundwater recharge (Fig. 3b) which are often important in triggering deep-seated landslides, are also observed, but to a lesser extent than changes in the extent of landslide-prone areas. Changes in both RCP scenarios are most pronounced in winter when between 6 and 8% more groundwater recharge is expected. There are only minor changes in groundwater recharge in the other seasons, except for the summer months, when there is a slight groundwater recharge decrease in the second projection period under RCP4.5 and a slight decrease in the third projection period under RCP8.5.

### Future alerts for shallow landslides

**Figure 4:**
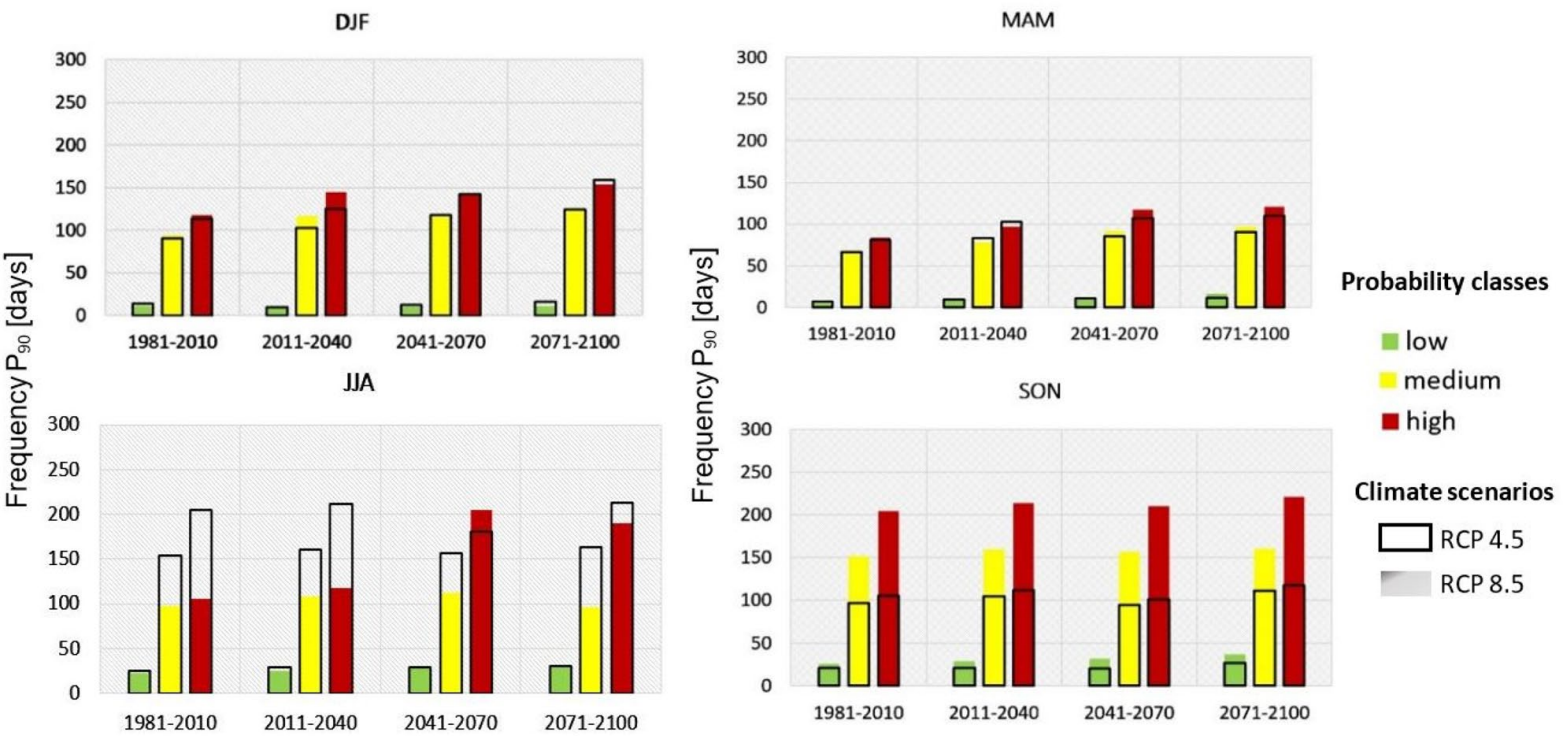
Frequency of landslide prediction alarms in days (1981 to 2100) associated with the 90 percentiles per three landslide probability classes (low, medium, high) for two climate scenarios (RCP 4.5 values are marked with transparent bars while RCP 8.5 are marked with coloured bars). The diagrams show the results for the baseline period and each future projection period separately.

**Figure 5:**
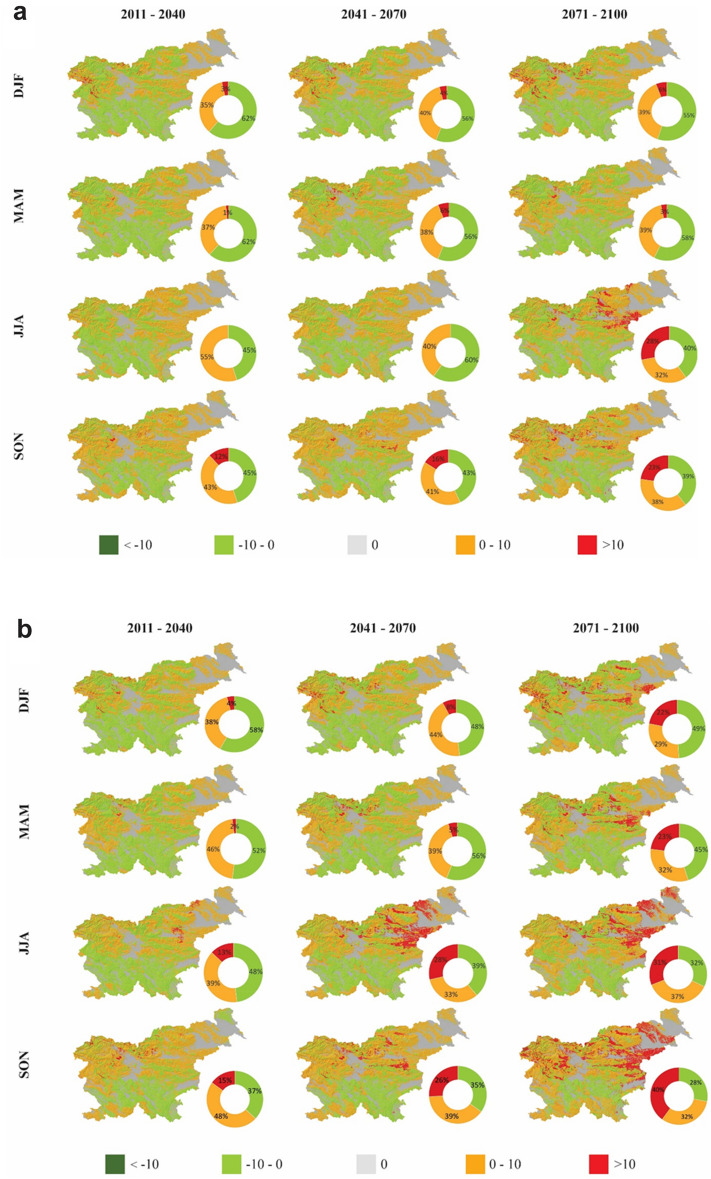
Spatial distribution of future shallow landslide alerts compared to the baseline period for (**a**) RCP4.5 and (**b**) RCP8.5 including pie charts indicating the percentage of area that will be affected by each warning level. Areas marked green have more alerts during the baseline period (i.e., dark green with more than 10 alerts and light green between zero and 10 alerts). Areas marked grey indicate no change in the number of alerts during the forecast periods compared to the baseline period. Areas colored orange and red indicate more alerts in the projection periods compared to the baseline period.

Further analysis of climate scenarios considers the MASPREM algorithm for calculating seasonal future alerts for shallow landslides following the workflow shown in Fig. 9. The results of the seasonal landslide prediction alerts for three landslide probability classes are presented with the 90th percentile in Fig. 4 for future projection periods and baseline periods separately. While Fig. 5 shows the spatial distribution of future shallow landslide alerts in the projection periods compared to the baseline period.

Comparing seasonal alerts and probability classes, the increase of alerts in the medium and high landslide probability areas is significant for climate scenario RCP8.5 in all seasons. The highest number of alerts (more than 200) is expected in the high landslide probability areas in summer and autumn in the middle and end of the century. In the case of climate scenario RCP4.5, an increase of landslide alerts is also expected in all seasons, but not as significant as in the case of RCP8.5. The highest number of warnings is expected for the summer months, especially for the areas with medium and high landslide probability in all observed projection periods, including the baseline period.

Figure 5 illustrates the spatial distribution of future alerts compared to the baseline period for RCP4.5 and RCP8.5 climate scenarios for the whole country. In the case of RCP4.5 (Fig. 5a), most alerts occur within zones of landslide-prone areas in the north-western part of the country, central Slovenia and eastern and northeastern parts of the country. The number of alarms increases from the present time to the middle of the century and towards the end of the century, where the largest number of alarms is expected in the spring and autumn. While in the northwestern part of the country (Alpine area), the highest alarm is expected in the winter. In the case of RCP8.5 (Fig. 5b), the trends are similar but more pronounced. Landslide alerts will affect larger areas.

## Discussion

For this study, we consider global climate simulations participating in the CMIP5 initiative which was found to be statistically significant in central Europe and the Mediterranean^42^. The latest IPCC report 2023^43^ also further warns that greenhouse gas emissions have continued to increase with widespread and rapid changes also in landscapes. For the countries of Central and Mediterranean Europe, where Slovenia is located, less rainfall and more extreme events such as heavy storms and short- intense rainfall are observed in summer. On the one hand, the seasonal variability of rainfall for the shallow and deep-seated landslides investigated in this study (Fig. 3) could be consistent with these results, because we observed that the most landslide-prone areas are expected in the winter season, followed by the summer season, which we could attribute to the more rainfall in winter and short intense rainfall in summer. In case of Fig. 4 the highest number of shallow landslide alerts is expected in summer for RCP4.5 and in autumn for RCP8.5. The results of Figs. 3 and 4 are both derived from the MASPREM algorithm, which determines landslide-prone areas based on a landslide susceptibility model calculated from lithology and data from DEM (slope, aspect, curvature) with a cell size of 12.5 m and contribute to the data quality. Therefore, both the climate change model and the methodology used have an impact on forecasted landslide prone areas. We are aware of the limitations of the MASPREM and mGrova algorithms, but in this case, we assume that the main weight of the results lies precisely in the input data of the selected climate model with a cell size of 1 km. However, there is a possibility that other conclusions could be drawn about the landslide areas alerted and affected in the future, also since the occurrence of landslides is also very much dependent on anthropogenic factors (human intervention in the landscape), which are not considered in the approach used in this study.

The largest changes to landslide prone areas are expected during winter months when the cyclon brings most of the rainfall in this region. Rainfall at the annual level and in winter under the RCP4.5 and RCP 8.5 scenarios increases significantly in the middle or end of the twenty-first century. An average increase of up to 20% is expected compared to the 1981–2010 baseline period, in case of RCP8.5 also up to 60% in winter. This rainfall increase is more pronounced in winter in the eastern part of the country what is also reflect in number of alarms in Fig. 4b in case of RCP8.5. The alerts signals are more significant for the easter parts of the country, where the highest numbers are expected in the autumn and summer, although the landslide-prone areas do not increase as much in winter, as shown in Fig. 3. The MASPREM alerts are however very much related to the summer heavy rainfall events with short duration but high intensity of rainfall. Such rainfall patterns are also increasingly observed in the last 10 years^44^ and are significant for shallow landslides also in neighbouring countries^3,45^. Inversely the spatio-temporal occurrence of deep-seated landslides cannot be unambiguously determined because their triggering also depends very much on local hydrogeological and tectonic conditions, which are not considered in MASPREM.

Such analyses are characterized by inherent errors in scenario-driven climate projections, and by epistemic uncertainties of the hydrological and slope stability modelling. Such uncertainties could be even larger in the case of analyses of deep-seated landslides, for which the effect of rainfall (ongoing and projected) is not direct and its analysis not trivial, as stated above. Several other variables, such as snowmelt, groundwater level, pore pressure, suction, etc. become relevant. However, regional hydrological analyses are be useful to reduce the modelling uncertainty, and to explain the general landslide activity in a region.

Overall, based on the results of this analysis, RCP4.5 indicates that at least up to 10 rainfall events could be expected to trigger shallow landslides over 40% of the area at mid to end century in all seasons. While more than 10 rainfall events are expected in summer and autumn over 27% of the area. In the case of the worst case scenario (RCP8.5), the expected climate change has greater impacts on the landscape. By mid-century, significantly more than 10 rainfall events are predicted for the summer and autumn months, which could affect more than 25% of the area. By the end of the century, the situation could be even worse. More than 10 rainfall events could also affect more than 40% of the area in summer and autumn. Although the landslide susceptibility map (Fig. 2a) classified 7% of the area as very high and 17% of the area as highly susceptible to landslides, these results showed that larger area could be affected by landslides by the end of the century. We are aware of the limitations of the MASPREM and mGrova algorithms, but in this case, we assume that the main weight of the results lies precisely in the input data of the selected climate model with a cell size of 1 km. However, there is a possibility that other conclusions could be drawn about the landslide areas alerted and affected in the future, also since the occurrence of landslides is also very much dependent on anthropogenic factors (human intervention in the landscape), which are not considered in the approach used in this study.

**Figure 6:**
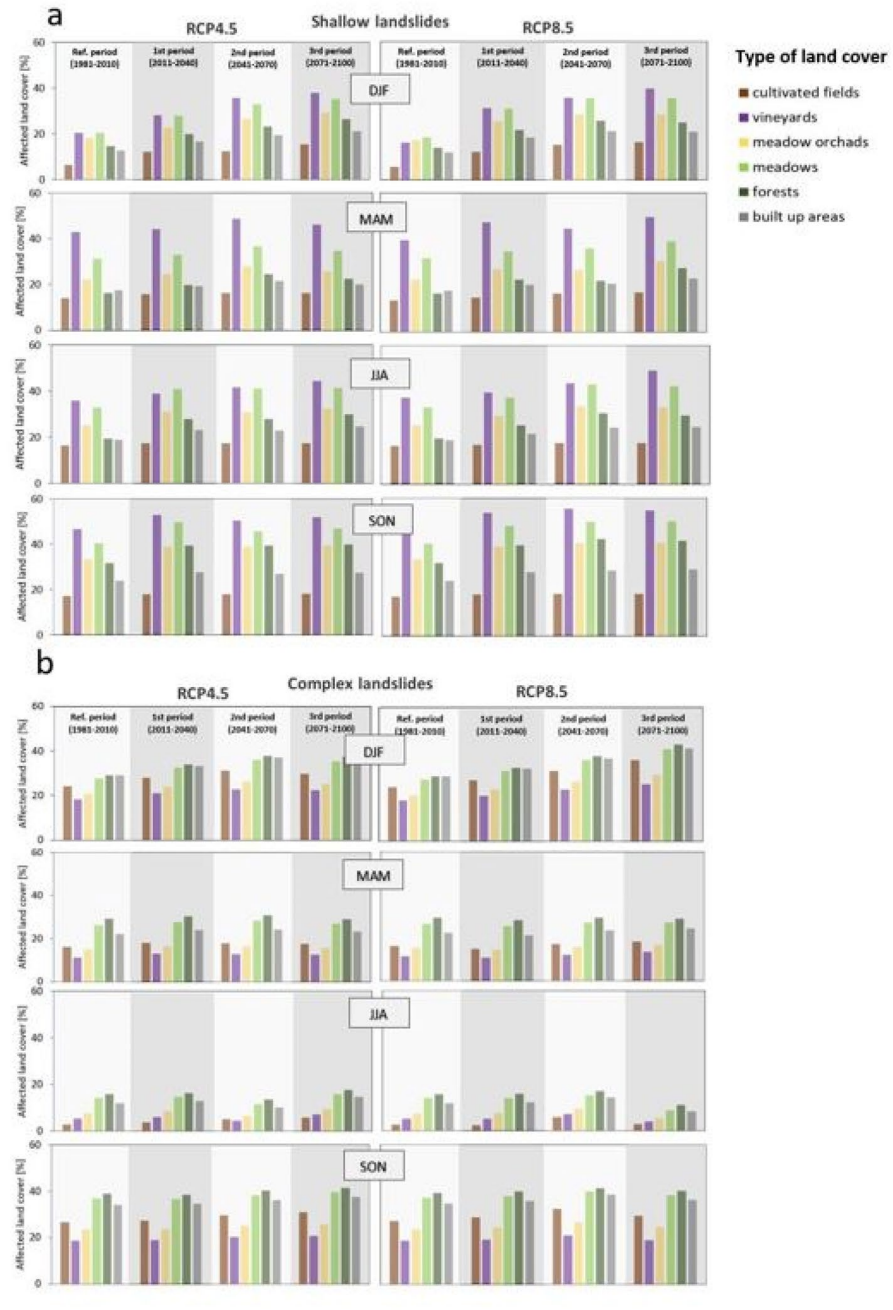
Land cover changes from 1981 to 2100 due to seasonal rainfall variability. Land cover changes are shown as percentages of land cover types in the 50th percentile values of all six climate models for the RCP4.5 and RCP8.5 climate scenarios. (**a**) impacted land cover for the shallow landslides. (**b**) impacted land cover for the deep-seated landslides.

To better understand effect of landslides on landscape development we performed statistical analyses of overlapping. Seasonal rainfall variability for shallow and deep-seated landslides area compared to the land cover and results are shown in Fig. 6. Results are presented as the percent of land cover types affected by seasonal rainfall variability in the 50th percentile values of all six climate models for the RCP4.5 and RCP8.5 climate scenarios. We considered six main classes of land cover in which landslides are most likely to occur so far (cultivated fields, vineyards, meadow orchards, meadows, forests, and built-up areas, shown in Fig. 2a).

In the case of shallow landslides (Fig. 6a) we show that in winter, spring, and summer areas with vineyards, meadows, and meadow orchads are mainly affected in all projection periods of both RCP4.5 and RCP8.5 climate scenarios, with percent between 20 and 40%, and even more in autumn. Forests, built-up areas and cultivated fields will be impacted less. Shallow landslides mostly affect soil of small thickness (generally less than 2 m) originating from the weathering of the bedrock (residual) and downslope transportation (colluvial)^46^. Figure 6b shows the results for deep-seated landslides. The largest changes in winter and autumn are expected for meadows, forests, and built-up areas in all projection periods, with percentages ranging from 20 and 40%, followed by cultivated fields and vineyards. Spring and summer are expected to have the lowest changes in groundwater levels, which translates into a lower percentage of affected land areas. Results suggest that shallow landslides will have a greater impact on the landscape in spring and summer than deep-seated landslides, while we can expect relatively similar effects of landslides on landscape in autumn and winter, where a very small difference is observed between RCP4.5 and RCP8.5 in all projected periods. However, both types have longer-term impacts on landscape change, but further analysis is needed to assess the dynamics of their impacts on landscape evolution on time scales of years to decades.

Comparing the number of shallow landslides per season by land cover class for the period between 1996 and 2022 (Fig. 2c), similar trends can be observed for both the RCP4.5 and RCP8.5 climate scenarios, which differ depending on the type of landslides observed. Shallow landslides (Fig. 6a) will occur mainly in vineyards, followed by meadows and orchards. Vineyards in Slovenia are often located on terraces (eastern, southwestern, and western parts of the country), which are man-made and consequently more prone to landslides^44,47^. The results of this study show similar trends in the projected periods as well. Deep-seated landslides (Fig. 6b), on the other hand, occur mainly in meadows, forests, and built-up areas. These trends are also consistent with the evidenced deep-seated landslides in Slovenia in the last 20 years, which is presented by Jemec Auflič et al.^41^. They found that deep-seated landslides in Slovenia are strongly influenced by deep-seated geological conditions, local hydrogeological conditions, and geomechanical properties of soils. Before adaptation measures are taken, the policy makers should consider that deep-seated and shallow landslides are inherently different in their characteristics.

## Conclusions

Changing climate at the junction of the Alps, the Pannonian Plain, the Dinaric Mountains, and the Mediterranean region will impact on landslides occurrences. Specifically, in Slovenia, which is characterized by three different climatic zones and different geomorphological and geological features, heavy rainfall has triggered numerous landslides in the last 25 years. Based on the results of seasonal rainfall changes, the following assumptions can be identified:

1. The seasonal rainfall variability trend for RCP8.5 shows that the largest changes are expected for winter, when up to 12% more landslide-prone areas are expected at mid- and end-century compared to the baseline period. For RCP4.5, the trend in winter, summer, and autumn months shows a gradual increase in landslide-prone areas from 5 to 12% from the near-term to the end of the century. Changes in groundwater recharge, which often plays an important role in triggering deep-seated landslides, are also observed, but to a lesser extent than changes in the extent of landslide-prone areas. Changes in both RCP scenarios are most pronounced in winter, when between 6 and 8% more groundwater recharge is expected.
2. The highest number of alerts (more than 200) is expected in the high landslide probability areas in summer and autumn in the mid- and end of the century in eastern part of the country. In the case of climate scenario RCP4.5, landslide warnings are also expected to increase in all seasons, but not as significantly as in the case of RCP8.5.
3. The results indicate that shallow landslides will have a greater impact on the landscape than deep-seated landslides in the spring and summer, while relatively similar landslide impacts on the landscape are expected in autumn and winter, with a very small difference between RCP4.5 and RCP8.5 in all projected time periods. However, both types have longer-term impacts on landscape change, but further analysis is needed to assess the dynamics of their impacts on landscape evolution on time scales of years to decades. Shallow landslides will occur mainly in vineyards, followed by meadows and orchards, while deep-seated landslides will occur mainly in meadows, forests, and built-up areas.
4. The future assumptions and changes shown in this study were derived from the global climate simulations of the CMIP5.

We acknowledge that the analyses are characterized by uncertainties related to both climate projections and landslide hydrological modelling. However, regional hydrological analyses such the one proposed in this work can be useful to reduce the modelling uncertainty, and to estimate variations in landslide activity in a wide region, with useful implications for the design of adaptation strategies.

## Methods

### Climate scenarios data

For this work, we selected the six regional climate models (RCMs) from the EURO-CORDEX project^48^, with the global climate simulations from CMIP5 (Coupled Model Intercomparison Project phase) driven by the six global circulation models (GCMs) (Table 1). The new CMIP6 also exists only for the European scale and shows on some differences for Central Europe (where Slovenia is mostly) (37). Of the two available spatial resolutions, i.e., 0.11° (12.5 km) and 0.44° (50 km), we considered the 0.11° spatial resolution with a regular 12.5 km grid with spacing between computational points^49^. Six models (Table 1) were selected from 14 combinations of GCMs and RCMs that differ as much as possible from each other while reflecting as closely as possible the measured values of past climate variables. All six models are considered equally reliable. For this study, we considered climate scenarios variable: the daily rainfall datasets of two Representative Concentration Pathways (RCP), namely RCP4.5 (mid-way) and RCP8.5 (worst-case) for the time window from 1981 to 2100. Daily rainfall data were downscaled from 12.5 km resolution to 1 km^50^. The downscaling of the data was performed daily for all six RCMs. To analyse future climate impact on landslides, the calculated models were divided into three 30-year projection periods: 1st period (near-term) between 2011 and 2040, 2nd period (mid-century) between 2041 and 2070, 3rd period (end of the century) between 2071 and 2100. To show the characteristics of seasonal variations, shorter periods within a year were considered, namely four meteorological seasons: winter (December, January, February; hereafter also DJF), spring (March, April, May; MAM), summer (June, July, August; JJA), and autumn (September, October, November; SON). Future projections represent a 30-year maximum rainfall from the 30-year baseline period in the past (1981–2010).

**Table 1:** Table of climate models (abbreviations provided by Slovenian Environment Agency^49^), abbreviations are derived from meteorological canters that prepared the data (e.g. DMI—Danish Meteorological Institute, KNMI-Netherlands Meteorological Institute, SMHI—Swedish Meteorological and Hydrological Institute, IPSL—Institute Pierre-Simon Laplace France).

Global climate model (GCM)	Regional climate model (RCM)	Model	RCP 4.5	RCP 8.5
CERFACS-CNRM-CM5	CCLM4-8-17	CCLM1*	x	x
MPI-ESM-LR	CCLM4-8-17	CCLM2*	x	x
EC-EARTH	HIRHAM5	DMI	x	x
IPSL-CM5A-MR	WRF331F	IPSL	x	x
HadGEM2-ES	RACMO22E	KNMI	x	x
MPI-ESM-LR	RCA4	SMHI	x	x

With * are marked the models named after CLMcom centre. The Global Climate Model (GCM) provided boundary conditions, and the Regional Climate Model (RCP) recalculated the data to a smaller scale (about 12.5 km).

### Downscaling technique

To overcome the large biases in climate models, a number of methods have been developed to correct the biases. In all methods, it is important to recognize that the quality of the observational data sets determines the quality of the bias correction^51,52^. For this purpose, we first interpolated the regional climate models in a grid of about 12 km into a 1 km grid with bilinear interpolation and then applied a bias correction. The bias correction was performed separately for each model cell and for each regional model. We used non-parametric quantile mapping (QM) using empirical quantiles. All calculations were performed in Toll R software, where we used the package “qmap”^53^. The baseline period for the bias correction method was daily data from 1981 to 2010. We compare the distribution of model data and measurements in the baseline period and evaluate the differences using the quantiles of this distribution. We use the estimated differences as correction factors of the model data for the future on the selected quantile. The correction factors were calculated for each day of the year using a mowing window. For precipitation, we used a sliding window of 61 days with 100 quantiles. All these correction factors were then applied to the future periods. After correcting for the biases, we analysed the trends of the extreme values using the generalized extreme value (GEV) distribution. We analysed the linear trend of the location parameter (mu0) using the R package “extRemes”^54^. The change in trend between the raw and the bias-corrected model data was not statistically significant, and the average values over the baseline period were also consistent with the measurements.

**Figure 7:**
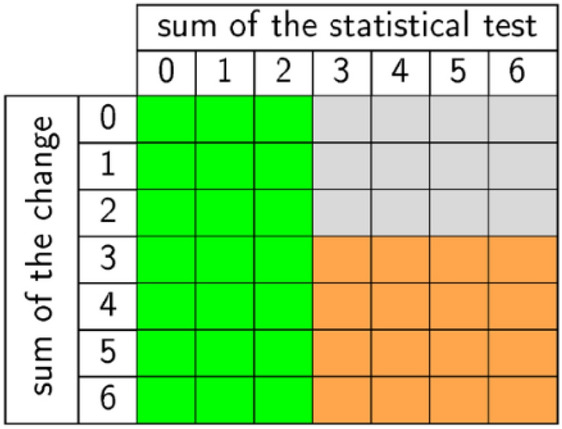
Matrix of our reliability test.

Our aim was to assess the changes in the number of days above a certain threshold (70 mm, 90 mm, 120 mm, 150 mm, 180 mm, 210 mm and 240 mm). For this purpose, we compared data of the projected periods (2011–2040, 2041–2070 and 2071–2100) with a baseline period (1981–2010). To evaluate the reliable changes of the ensemble model, we used the out-test (Fig. 7). For each model cell, we calculated the sum of the signs of the changes and the sum of the statistical changes. If all models show statistically significant changes and these changes are all in the same direction, we indicated this change as a reliable change (orange color). If less than half of our models show a statistically significant change, then there is no change (green color). However, if most of the models show a statistically significant change, but some of them show an increase in days above the threshold and some will have decrease in these days, this change is unreliable (gray color).

**Figure 8:**
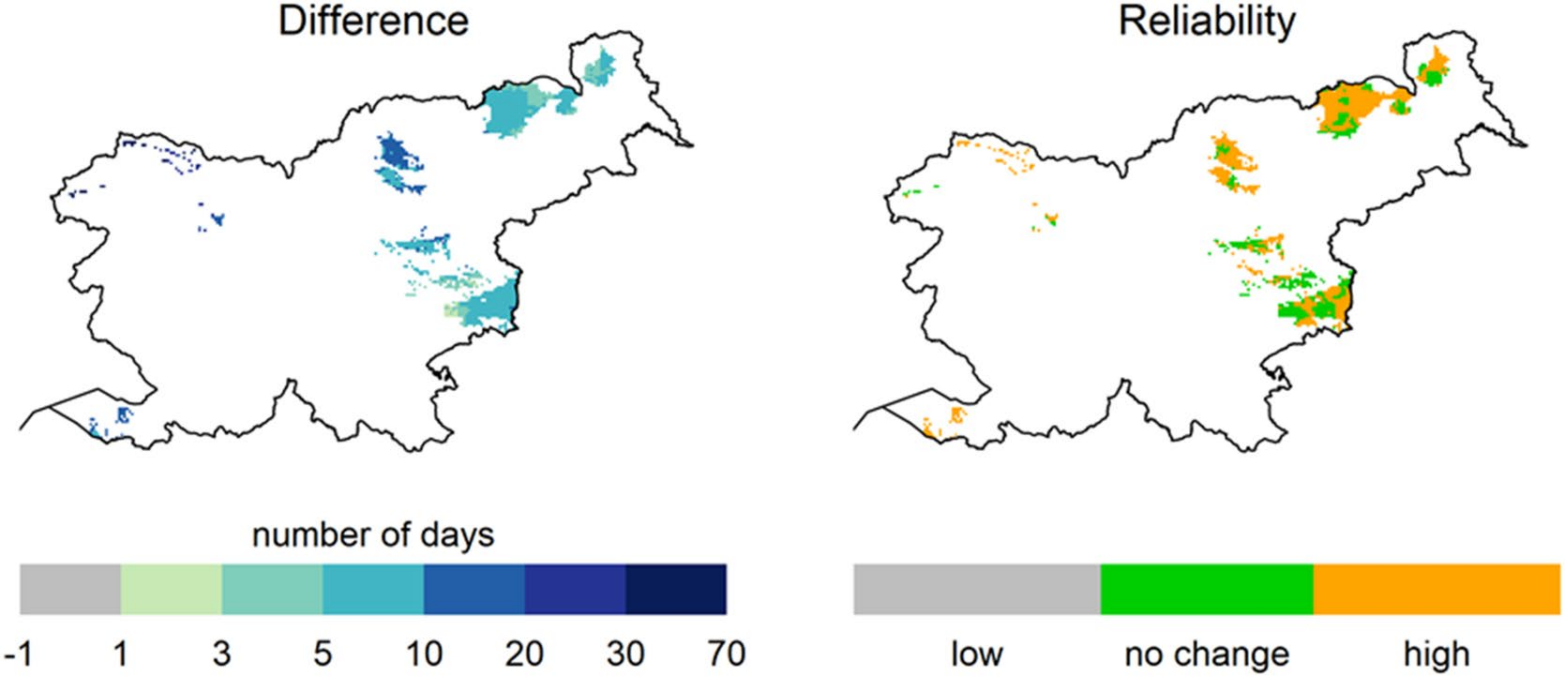
Example of number of days above 70 mm rainfall threshold in the period 2071 and 2100.

Figure 8 shows the model mean of the differences in the number of days above specific threshold and the corresponding reliability test. Orange colour represents model cells where we expect high reliability of change in the future, and green colour represents model cells with no changes. However, this doesn’t mean that there will be no changes, but the changes will be less than the natural variability of the data.

### Defining calculation models for shallow landslides

**Figure 9:**
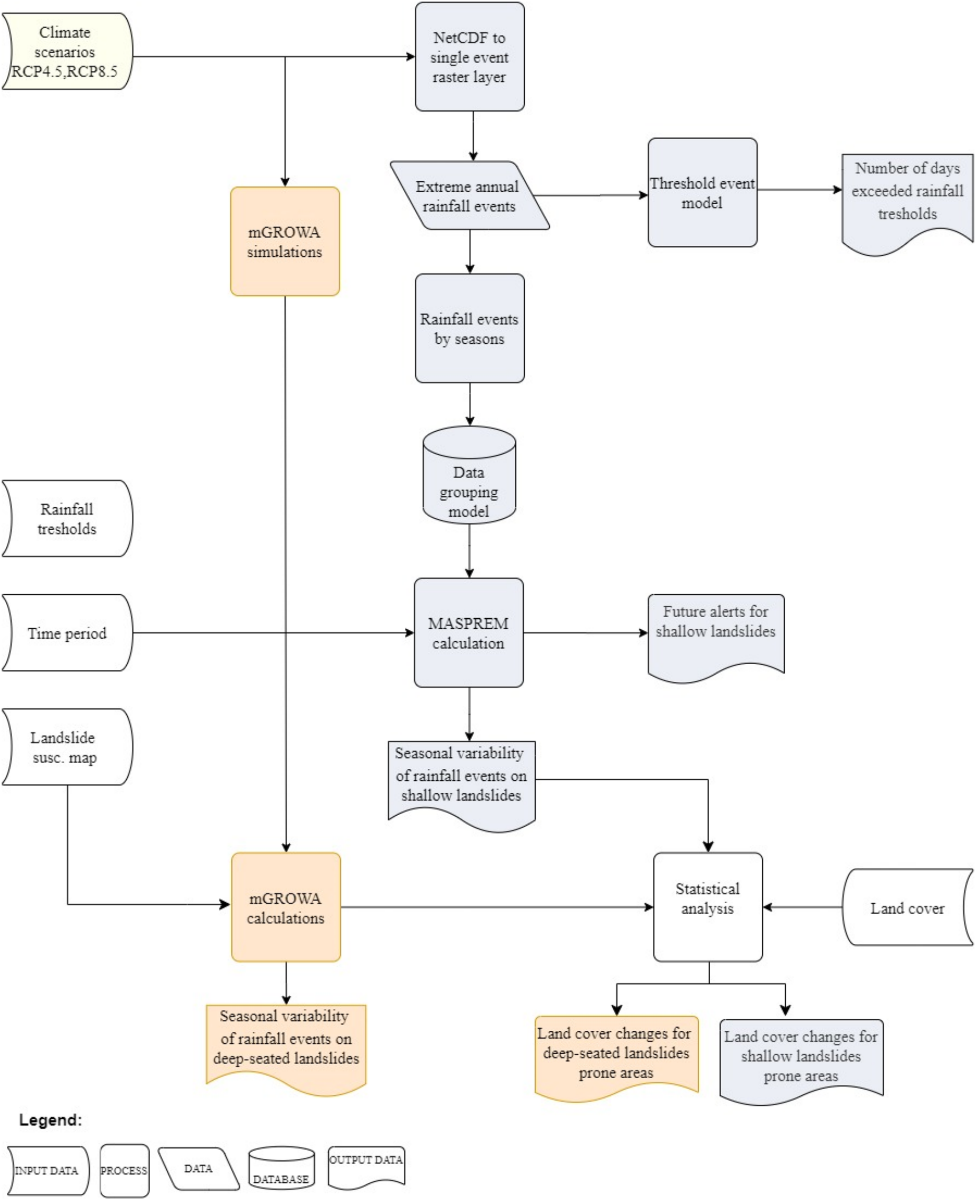
Workflow for defining computational models for shallow landslides with the MASPREM algorithm (blue process steps) and for deep-seated landslides with mGrova (orange process steps).

The impact of future seasonal rainfall variations on landslides and the impact on land cover changes were calculated in three phases according to the workflow diagram shown in Fig. 9. In the first phase, the time series climate models were separated into individual raster using ArcGIS set tool "Make NetCDF raster layer”, based on which the extreme annual rainfall events were defined for the individual raster cell (Fig. 9). To determine the rainfall values for extreme annual events, first, the maximum annual rainfall for a single cell for all three projection periods and baseline periods was calculated. The rainfall values are overlaid with shallow landslide rainfall triggering values as part of the algorithm developed by Komac et al.^55^ and Jemec Auflič et al.^38^, which determines the areas where the rainfall thresholds are exceeded and the degree of exceedance. In the case of shallow landslide occurrences in Slovenia in 1996 and 2022, the maximum rainfall threshold, defined as the level above which a shallow landslide always occurs^56^ is 70 mm (Fig. 2d), especially for the engineering geological (EG) units where clayey, slaty clays, marls and scree components predominate. The cells representing areas with exceeded rainfall threshold have a value of 1, all other areas were set to 0. From these grids, we created a threshold event model that determined the number of days with exceeded rainfall thresholds based on rainfall thresholds and extreme annual rainfall events. The main purpose was to determine the difference in the number of days with exceeded rainfall values in the projection periods (2011–2040, 2041–2070, 2071–2100) compared to the baseline period (1981–2010). This also gave us an overview of the number of extreme events (whether there will be only one extreme event or several) and their spatial distribution.

In the second phase, we estimated the seasonal variability of rainfall and the effects on shallow landslides by the end of the twenty-first century. Extreme annual rainfall events were sorted by seasons and combined into 30-year maximum rainfall events using the data grouping model (Fig. 9). To assess the seasonal variability of rainfall on landslides (Fig. 3), we applied an algorithm developed for Slovenian national landslide forecasting system (MASPREM^38,55^). The system predicts rainfall-induced landslides on five level warning scale using a fuzzy logic algorithm described in Jemec Auflič et al.^38^ based on the 1:250,000 scale landslide susceptibility map, rainfall thresholds and rainfall forecast model (ALADIN/INCA). In this study, 30 years of maximum rainfall events were used as a forecast model and replaced the rainfall forecast models used in the MASPREM system. The algorithm is running in a Python script and the results in the form of grids for all six climate models represent the areas where an increased probability of landslides due to changes in rainfall is anticipated. Since each of the six models presented in Table 1 must be considered equally reliable or equally unreliable^49^, we present the results in terms of the percentage of area in the landslide susceptibility class as the median or percentiles 25 and 75 of all six models combined. The alerts for shallow landslides included all model computations that exceeded the maximum precipitation threshold and all model computations from the low probability class. For this study, we introduced three classes for the probability of landslides (low, medium, high). The following statistical parameters were provided for each calculated model: Minimum, Maximum, Range, Mean, Standard Deviation, Sum, Median, and Percentiles 10 and 90. In the final phase (Fig. 9), we performed statistical overlap analyses to determine the impact of alerts on land cover, using Corine land cover data from 2018 (https://land.copernicus.eu/pan-european/corine-land-cover/clc2018; last accessed 13/03/2023) and cadastral data on actual agricultural and forest land cover from Slovenia (GERK). We calculated the percent area of all six climate models RCP4.5 and RCP8.5 for land cover types where landslide alerts for shallow landslides occur, including geologic settings and slope inclination.

### Defining calculation models for deep-seated landslides

Water balance model mGROWA was developed from older GROWA model^57,58^ in Germany and was applied to Slovenia by Frantar et al.^59^. mGROWA is a deterministic water balance model, calculating runoff generation and runoff components including net groundwater recharge in daily and monthly time steps. Model grid resolution is 100 m^60^. Compared to GROWA model the mGROWA uses improved soil module and newly developed snow module, beside that the calculating interval is daily compared to annual GROWA time-step. The basic model concept is soil hydrology and runoff generation. The precipitation is the main input of the water for the simplified multi-layer soil moisture model that calculates transpiration and percolation of water in a grid cell with parameters derived from GIS layers. The storage part of water balance is defined differently for different site conditions. One of the most important parameters of the soil module are crop coefficients derived from literature and field experiment data. The calculated total runoff is separated within the model into direct runoff and groundwater recharge, where the groundwater recharge corresponds to the base flow over longer periods.

Based on the conducted mGROWA climate change simulations^61^ we decided to use the “qrn” variable, which represents the groundwater recharge, as a proxy for triggering the deep-seated landslides. The reason for this selection was because groundwater is a particularly important parameter in triggering deep-seated landslides^62^. Hence, the basic assumption was that the increased or decreased groundwater recharge as part of the water-balance mGROWA model would lead to increased or decreased deep-seated landslides activity, respectively. The area of Slovenia was divided into three sub-areas based on the landslide susceptibility map. Additionally, some comparison in the groundwater recharge was conducted for the entire area of Slovenia. For each probability class (i.e., low, medium and high landslides probability) the average groundwater recharge (i.e., “qrn” variable) was calculated for all the models listed in Table 1 and for four seasons. Furthermore, a similar methodology as described in Fig. 9 was used for the comparison of the future with the baseline scenario and also for the investigations related to the land cover data.

## Data Availability

The datasets generated during and/or analysed during the current study are available from the corresponding author.
